# Docosahexaenoic Acid Enhances Oxaliplatin-Induced Autophagic Cell Death via the ER Stress/Sesn2 Pathway in Colorectal Cancer

**DOI:** 10.3390/cancers11070982

**Published:** 2019-07-14

**Authors:** Soyeon Jeong, Dae Yeong Kim, Sang Hee Kang, Hye Kyeong Yun, Jung Lim Kim, Bo Ram Kim, Seong Hye Park, Yoo Jin Na, Min Jee Jo, Yoon A. Jeong, Bu Gyeom Kim, Dae-Hee Lee, Sang Cheul Oh

**Affiliations:** 1 Department of Oncology, Korea University Guro Hospital, Korea University College of Medicine, Seoul 08308, Korea; 2 Graduate School of Medicine, College of Medicine, Korea University, Seoul 08308, Korea; 3 Department of Surgery, Korea University Guro Hospital, Korea University College of Medicine, Seoul 08308, Korea

**Keywords:** Oxaliplatin, docosahexaenoic acid, Sestrin 2, autophagic cell death, colon cancer

## Abstract

Oxaliplatin is an anticancer drug administered to colorectal cancer (CRC) patients in combination with 5-fluorouracil and antibodies (bevacizumab and cetuximab), thereby significantly improving the survival rate of CRC. However, due to various side effects associated with the above treatment strategy, the need for combinatorial therapeutic strategies has emerged. Based on the demand for new combinatorial therapies and the known antitumor effects of the omega-3 polyunsaturated fatty acid, docosahexaenoic acid (DHA), we investigated the Oxaliplatin and DHA combination for its effect. Our results indicated that DHA further enhanced Oxaliplatin-induced cell viability and autophagic cell death, in vitro and in vivo. Oxaliplatin and DHA also increased the expression of Sestrin 2 (SESN2) and endoplasmic reticulum (ER) stress related C/EBP homologous protein (CHOP). Additionally, treatment with Oxaliplatin and DHA enhanced the binding of CHOP to the promotor region of SESN2, increasing SESN2 expression. These results suggested that DHA enhanced Oxaliplatin-induced reduction in cell viability and increase in autophagy via activating SESN2 and increasing ER stress. Thus, SESN2 may be an effective preclinical target for CRC treatment.

## 1. Introduction

Colorectal cancer (CRC), which is one of the most common gastrointestinal cancers, is associated with a high mortality rate [1]. Currently, chemotherapy for CRC patients is generally performed using bevacizumab in combination with FOLFOX (5-fluorouracil (5-FU) and leucovorin with Oxaliplatin) or FOLFIRI (5-fluorouracil, leucovorin with irinotecan), rather than alone with the anticancer drugs [2]. Oxaliplatin is the first platinum-based compound proven to be effective against CRC. It forms covalent bonds with DNA, which inhibits DNA replication and transcription, and induces cell death [3]. Additionally, Oxaliplatin has been reported to induce autophagy, as well as apoptosis [4,5].

Autophagy (macroautophagy), which regulates responses to various stresses such as starvation and oxidative stress, is considered to be an important mechanism that maintains cellular homeostasis [6]. Upon activation of autophagy, a phagophore swallows intracellular organelles and cellular proteins, and it elongates the cell membrane to form an autophagosome, which then fuses with lysosomes for degradation and recycling of the swallowed organelles and proteins [7]. In response to stress, autophagy is regulated by many pathways, among which the AKT/mammalian target of the rapamycin (mTOR) signaling pathway inhibits autophagy most strongly [8]. AKT/mTOR signaling regulates cell proliferation, survival, and various intracellular metabolism. Sestrin (SESN) is a negative regulator that modulates mTOR by activating 5′ AMP-activated protein kinase [9,10]. SESN is a highly conserved metabolic regulator, which is activated under stress conditions, such as DNA damage, starvation, endoplasmic reticulum (ER) stress and oxidative stress. SESN proteins are divided into three subtypes, SESN1, SESN2, and SESN3, of which SESN2 is the most potent autophagy inducer [11]. SESN2 upregulation has been associated with autophagy in neuroblastoma SH-SY5Y cells and bladder cancer UMUC3 cells [12,13].

Docosahexaenoic acid (DHA), an omega-3 polyunsaturated fatty acid (ω3-PUFA), is an essential fatty acid with its first double bond at the ω3 position [14]. DHA has shown anticancer effects in several types of cancers [15,16,17]. The mechanisms underlying the anticancer effect of DHA reportedly include regulation of Wnt/β-catenin inhibition [18], oxidative DNA damage [19], and mitogen-activated protein kinase activation [14]. Furthermore, our previous studies have revealed that DHA induces autophagy whilst suppressing mTOR in human cervical cancer, prostate cancer, lung cancer, and glioblastoma cells [20,21,22,23]. Although DHA has been reported to exert an anticancer effect on colorectal cancer via autophagy, it is not yet certain whether DHA induces autophagy via SESN2 activation.

In this study, we investigated whether DHA further enhanced autophagy induced in colorectal cancer cells by Oxaliplatin. Our results indicated that DHA further promoted Oxaliplatin-induced cell viability reduction and autophagy induction, via ER stress induction and SESN2 activation. Considered together, our data suggest that DHA-induced autophagic cell death plays an important role in the improvement of Oxaliplatin-induced autophagy via ER stress overproduction and SESN2 upregulation. Our study presented clinical evidence indicating that the Oxaliplatin and DHA combination showed potential as a novel therapeutic strategy for CRC treatment.

## 2. Results

### 2.1. Oxaliplatin and DHA Reduces Viability and Induces Cell Death in Human CRC Cells

**Figure 1:**
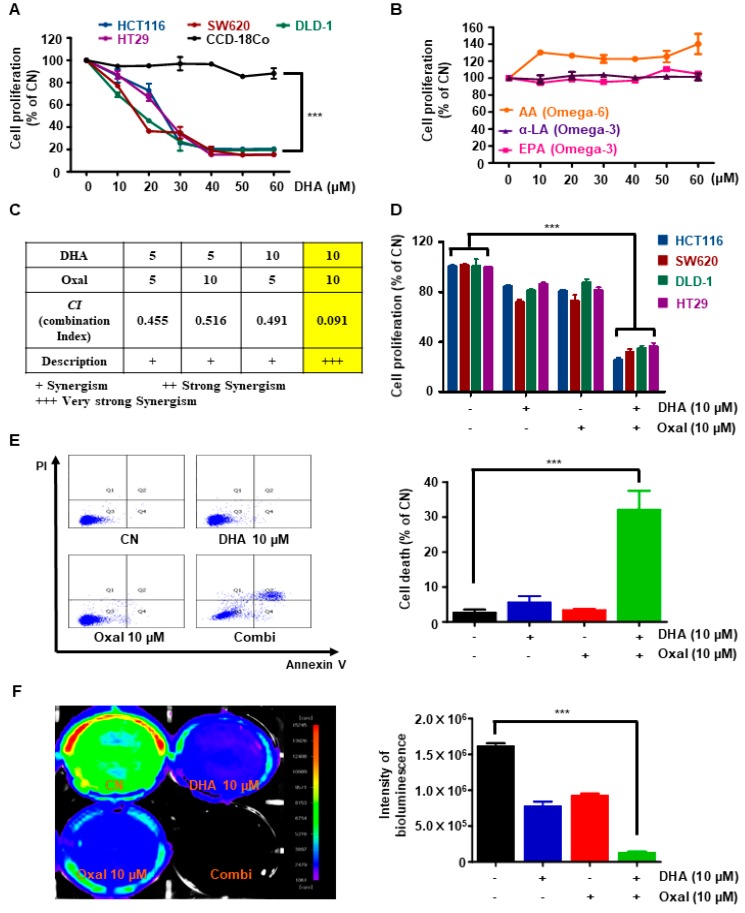
Combinatorial treatment with Oxaliplatin and docosahexaenoic acid (DHA) reduces viability and induces cell death in human colorectal cancer (CRC) cells. (**A**) Human normal colon CCD-18Co cells and various human CRC cells were treated with 0–60 µM of DHA for 24 h. Cell viability was measured via a WST-1 assay. ***, *p* < 0.001; (**B**) HCT116 cells were treated with 0–60 µM of ω3-polyunsaturated fatty acids (PUFAs) or ω6-PUFAs for 24 h. Cell viability was determined via a WST-1 assay; (**C**) Combination index (CI) for Oxaliplatin and DHA; (**D**) CRC cells were treated with the indicated doses of Oxaliplatin and DHA for 24 h. Cell viability was determined via the WST-1 assay. ***, *p* < 0.001; (**E**) Cell death was measured via Annexin V/propidium iodide staining using flow cytometry in HCT116 cells. ***, *p* < 0.001; (**F**) Intensity of bioluminescence after treatment with Oxaliplatin and DHA in the HCT116 Luc^+^ cells. The captured images were quantified using Image J. ***, *p* < 0.001.

First, to elucidate the cytotoxicity of DHA in CRC cells, various CRC and normal colon cells were cultured with different concentrations (0–60 μM) of DHA for 24 h. DHA decreased the viability of CRC cells in a dose-dependent manner, but not in normal colon CCD-18Co cells (Figure 1A). To investigate whether other PUFAs also inhibited the viability of CRC cells, we treated the cells with ω6-PUFAs arachidonic acid (AA) and other ω3-PUFAs (alpha-linolenic acid (α-LA), and eicosapentaenoic acid (EPA)). Interestingly, AA, α-LA, and EPA did not affect the viability of the CRC cells and had no combined effect with Oxaliplatin (Figure 1B, and Figure S1A–C).

To determine whether the Oxaliplatin and DHA combination exerted synergistic effects, a combination index (CI) was calculated. A combination of Oxaliplatin 10 μM and DHA 10 μM showed the strongest synergistic effect (Figure 1C). Moreover, the above doses of Oxaliplatin and DHA treatment in various CRC cells significantly reduced cell viability and elevated the number of Annexin V/propidium iodide (PI) double-stained cells compared to single treatments with each agent (Figure 1D,E). Oxaliplatin and DHA combination-induced cell death was confirmed via in vivo bioluminescence imaging. Oxaliplatin and DHA decreased bioluminescence intensities (Figure 1F), indicating that DHA combination with Oxaliplatin increased cell death.

### 2.2. Oxaliplatin and DHA Combination Enhances Oxaliplatin-Induced Autophagy in Human CRC Cells 

**Figure 2:**
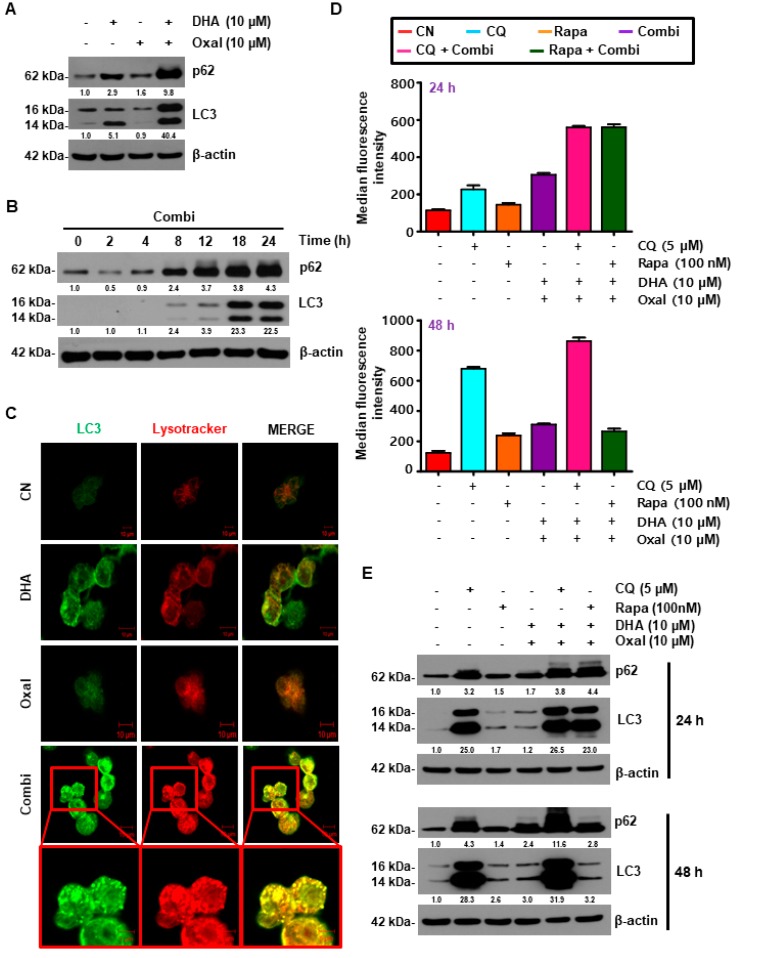
DHA enhances Oxaliplatin-induced autophagy in human CRC cells. (**A**,**B**) HCT116 cells were subjected to indicated doses (**A**) and time (**B**) periods for 24 h. Then, the protein level of microtubule-associated protein 1A/1B-light chain 3 (LC3) and p62 were analyzed by immunoblotting. (**C**) Formation of green fluorescence protein (GFP)-LC3 puncta following exposure to Oxaliplatin and DHA was analyzed using confocal microscopy (Scale Bar, 10 μm). (**D**,**E**) HCT116 cells were treated with Oxaliplatin and DHA in the absence or presence of chloroquine (CQ) or rapamycin for 24, and 48 h. Autophagic cells (**D**), and autophagic markers (**E**) were detected using immunoblotting and a flow cytometer with an autophagy detection kit.

To clarify whether Oxaliplatin and DHA-induced cell death could be attributed to autophagy, we evaluated HCT116 cells treated with Oxaliplatin and DHA for autophagy. The Oxaliplatin and DHA combination caused a dose- and time-dependent increase in the fluorescence intensity of p62, as well as in the protein expression of p62 and microtubule-associated protein 1A/1B-light chain 3 (LC3) (Figure 2A,B, and Figure S2A), which are widely used markers of autophagy [24]. This observation was also confirmed in cells that were virally infected with green fluorescence protein (GFP)-LC3, which revealed a higher number of GFP-LC3 puncta than the control cells following treatment with the Oxaliplatin and DHA combination (Figure 2C). To examine the effect of Oxaliplatin and DHA during autophagic flux, the HCT116 cells were exposed to Oxaliplatin and DHA, with or without rapamycin (autophagy inducer) and chloroquine (CQ, lysosomal acidification inhibitors) for 24 h. At 24 h, the number of autophagic cells and the expression of p62, as well as LC3 were both enhanced by rapamycin and CQ, whereas at 48 h, the number of autophagic cells and the expression of p62 and LC3 by CQ was significantly increased. Moreover, the autophagic cells and p62, and LC3 by rapamycin were not different from the combination of DHA and Oxaliplatin (Figure 2D,E, and Figure S2C). Additionally, the number of LC3 puncta, as well as the levels of autophagic markers, were also increased by the combined treatment of DHA and Oxaliplatin in other CRC cells, as well as in DLD-1 and SW620 cells (Figure S2B,D), suggesting that autophagy was increased, which indicated its involvement in Oxaliplatin and DHA combination-induced cell death.

### 2.3. SESN2 Is Associated with Autophagy Induced by Oxaliplatin and DHA Combination in Human CRC Cells

**Figure 3:**
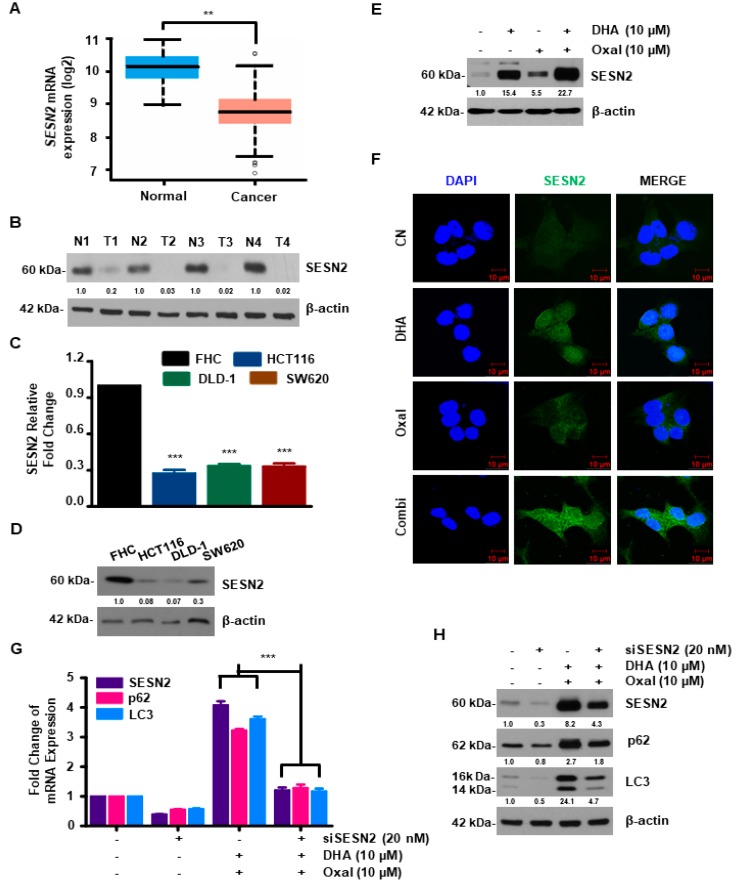
Combinatorial treatment upregulates expression of SESN2 in human CRC. (**A**) mRNA expression levels of SESN2 in normal and tumor tissues were evaluated using the mRNA expression data of the IlluminaHiSeq of TCGA CRC. **, *p* < 0.01. (**B**) SESN2 protein levels in normal and tumor tissues were evaluated using western blotting. (**C**,**D**) Comparison of endogenous expression levels of SESN2 mRNA and protein in normal human cells and human CRC cells using quantitative real-time polymerase chain reaction (qRT-PCR) analysis (**C**), immunoblotting (**D**). ***, *p* < 0.001. (**E**,**F**) SESN2 protein levels in the Oxaliplatin and DHA combination was analyzed by immunoblotting (**E**) and immunofluorescence (**F**) (Scale bar, 10 μm). (**G**,**H**) Following transfection with control small interfering RNA (siRNA) or SESN2 siRNA, cells were exposed to the Oxaliplatin and DHA combination. The mRNA (**G**) and protein (**H**) expression of LC3 and p62 were evaluated using qRT-PCR and western blotting. ***, *p* < 0.001.

SESN2 is known to play an important role in autophagy, as well as in colon carcinogenesis [11,25]. To this end, the relationship between SESN2 and human CRC was examined using the TCGA dataset. As shown in Figure 3A, the mRNA expression of *SESN2* was lower in the CRC tissue compared to solid normal tissue. Moreover, protein expression of SESN2 was significantly decreased in tumor tissues compared with normal tissues (Figure 3B). Furthermore, the mRNA and protein levels of SESN2 in CRC cells were diminished compared to normal colon FHC cells (Figure 3C,D). Western blotting and immunocytochemistry were performed to unveil the effect of SESN2 on the autophagy mechanisms induced by the Oxaliplatin and DHA combination. Protein and fluorescence intensity of SESN2 were higher in cells exposed to Oxaliplatin and DHA (Figure 3E,F). HCT116 cells were transfected with SESN2-specific small interfering RNA (siRNA). SESN2 knockdown partially decreased Oxaliplatin and DHA-induced autophagy (Figure 3G,H), indicating the link between Oxaliplatin and DHA-induced SESN2 activation and autophagic cell death.

### 2.4. DHA Promotes Oxaliplatin-Induced Autophagy via ER Stress

**Figure 4:**
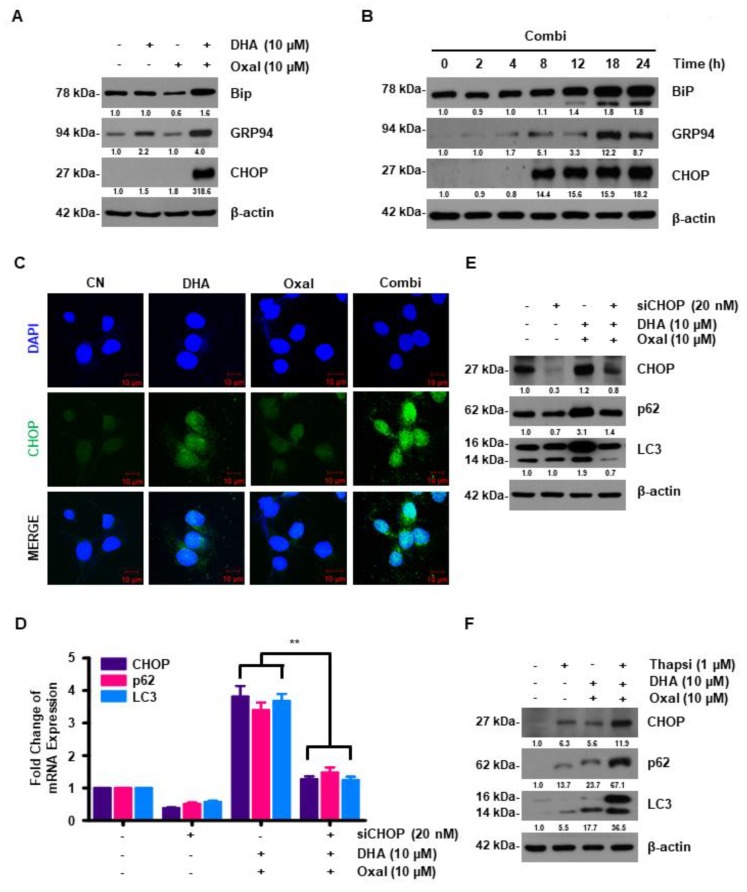
Combined treatment with Oxaliplatin and DHA induces endoplasmic reticulum (ER) stress overproduction. (**A**,**B**) HCT116 cells were exposed to different doses of Oxaliplatin and DHA (**A**) and time periods (**B**). HCT116 cell lysates were analyzed with an immunoblotting assay using ER stress-related antibodies. (**C**) C/EBP homologous protein (CHOP) was confirmed via immunofluorescence in the Oxaliplatin and DHA combination (Scale Bar, 10 μM). (**D**,**E**) Following transfection with control siRNA or CHOP siRNA, the mRNA(**D**) and protein (**E**) levels of LC3 and p62 were detected via qRT-PCR and immunoblotting. **, *p* < 0.001. (**F**) Cells were exposed to Oxaliplatin and DHA with or without thapsigargin, and the p62 and LC3 levels were observed via western blotting.

Autophagy and ER stress are closely related [11]. HCT116 cells were treated with Oxaliplatin and DHA, and we measured the protein levels of unfolding protein response (UPR) and C/EBP homologous protein (CHOP, a transcription factor of ER stress) via western blotting and immunocytochemistry. The Oxaliplatin and DHA combination caused a dose- and time-dependent increase in the fluorescence intensity of CHOP, as well as in the protein expression of both the UPR (glucose-regulated protein (GRP94) and binding immunoglobulin protein (Bip)) and CHOP (Figure 4A–C). To identify the regulation of autophagy by CHOP, we downregulated CHOP using CHOP-specific siRNAs. CHOP knockdown attenuated both the mRNA and protein levels of p62 and LC3 in HCT116 cells (Figure 4D,E). To confirm that autophagy induction in Oxaliplatin and DHA-treated CRC cells was caused by increased ER stress, thapsigargin, an ER stress inducer, was exogenously added to the HCT116 cells. Thapsigargin increased the mRNA and protein levels of p62 and LC3 (Figure 4F), indicating that ER stress regulated Oxaliplatin and DHA-induced autophagy. 

### 2.5. Oxaliplatin and DHA Combination-Induced CHOP Activation Regulates SESN2 in Human CRC Cells

**Figure 5:**
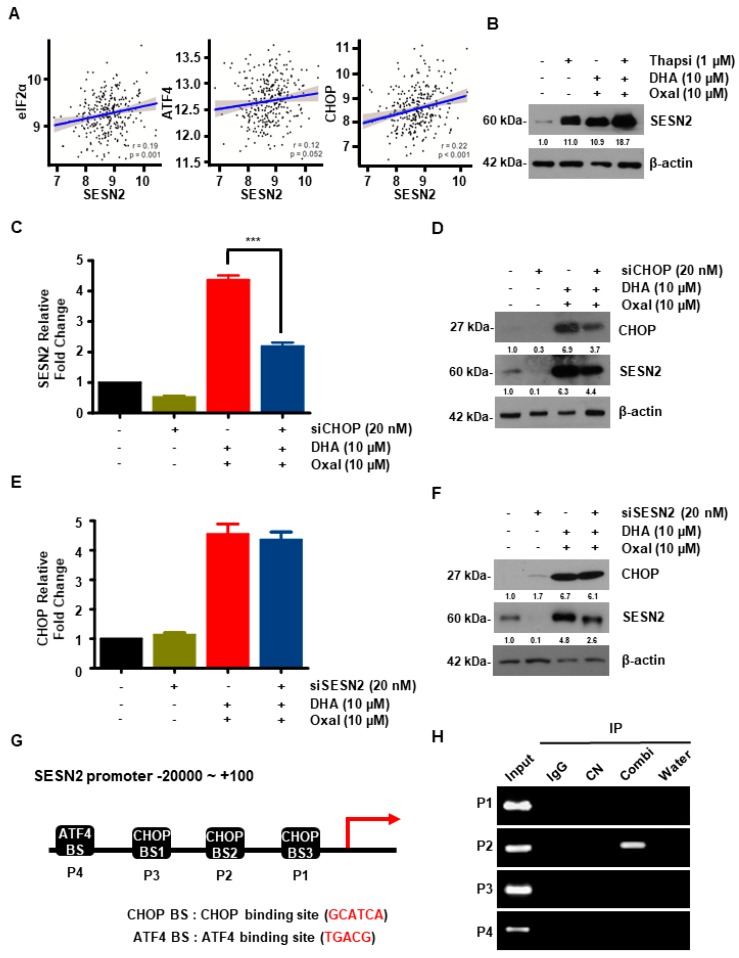
CHOP regulates SESN2 activity by binding directly to the SESN2 promoter region. (**A**) The correlation of SESN2 expression and ER stress-related genes in CRC patients. (**B**) HCT116 cells were pre-treated with thapsigargin for 1 h and then exposed to the Oxaliplatin and DHA combination for 24 h. SESN2 protein expression was confirmed with western blotting. (**C**–**F**) Following transfection with control siRNA, CHOP siRNA, or SESN2 siRNA, the SESN2 and CHOP protein levels were determined using qRT-PCR (**C**,**E**) and immunoblotting (**D**,**F**). ***, *p* < 0.001. (**G**) Illustration of the three predicted CHOP binding sites (BS) and ATF BS in the SESN2 promoter. (**H**) Cells were treated with Oxaliplatin and DHA, and then immunoprecipitated with either IgG or CHOP.

To investigate the correlation between SESN2 and the ER stress-related gene (eukaryotic translation inhibition factor 2A (eIF2α), activating transcription factor 4 (ATF4)**,** and CHOP) in CRC patients, we used a TCGA dataset. The mRNA expression of SESN2 was positively correlated with ER stress in the CRC patients of the TCGA dataset (Figure 5A). To ensure our above findings, we first treated thapsigargin (ER stress inducer) to the CRC cells. SESN2 protein levels that were increased by the Oxaliplatin and DHA combination were further increased by thapsigargin (Figure 5B). The cells were then transfected with CHOP and SESN2 siRNAs. CHOP knockdown reversed the increase in the SESN2 mRNA and protein levels, whereas the SESN2 knockdown did not affect CHOP levels (Figure 5C–F). Since SESN2 is known to be regulated by *ATF4*, an ER stress-related gene [26], we performed a chromatin immunoprecipitation (ChIP) assay to determine whether SESN2 activation was regulated by ATF4 or CHOP. The Oxaliplatin and DHA combination increased the binding of CHOP to the SESN2 promoter, but not ATF4 (Figure 5G,H). These findings indicated that CHOP directly bound to the SESN2 promoter region, resulting in the Oxaliplatin and DHA combination induced SESN2 upregulation.

### 2.6. Oxaliplatin and DHA Combination Induces Autophagy In Vivo

Two PDC cell lines were used to confirm the results obtained. The Oxaliplatin and DHA combination diminished the viability and elevated the mRNA levels of autophagy related genes (LC3 and p62), as well as SESN2 (Figure S3A,B).

**Figure 6:**
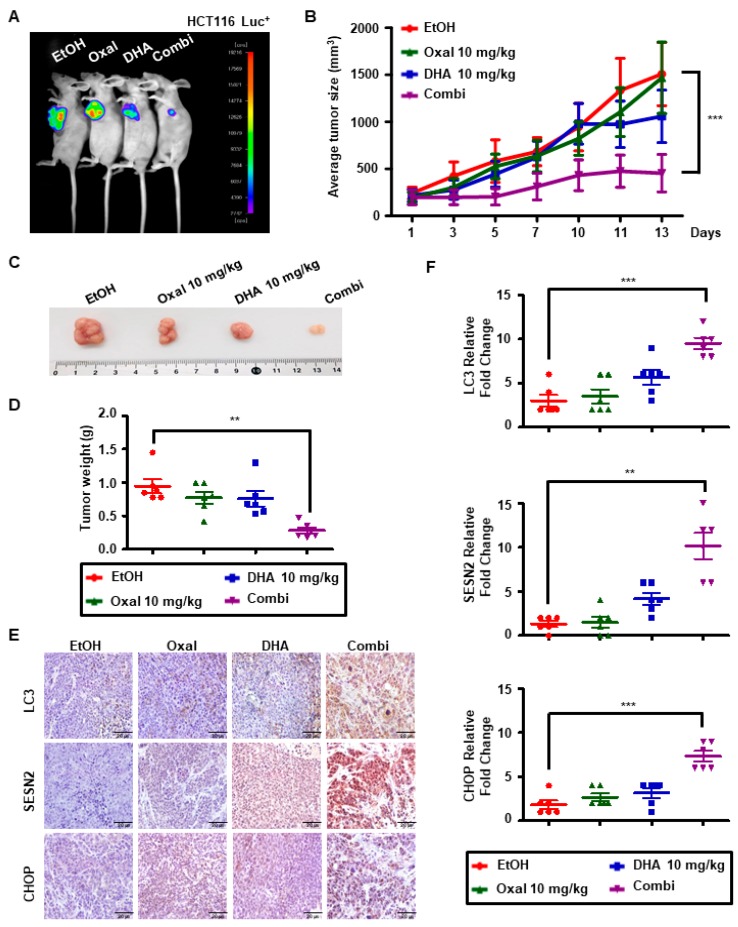
Combinatorial treatment with DHA and Oxaliplatin increases CHOP expression and in vivo autophagy. (**A**–**D**) Nude mice were subcutaneously inoculated with 1 × 107 HCT116-Luc+ Cells. When tumor volume reached approximately 200 mm^3^, the mice were treated with EtOH, DHA (10 mg/kg), Oxaliplatin (10 mg/kg), or the combinatorial treatment of DHA and Oxaliplatin (10 mg/kg), administered 3 times a week for 2 weeks. (**A**) Mice were imaged using the NightOWL LB983 bioluminescence imaging (BLI) system. (**B**) Line graph illustrating the tumor volume (mm^3^). ***, *p* < 0.001. (**C**) Tumor tissues were harvested on day 14 and then imaged using a digital camera. (**D**) Graph illustrating body weight. **, *p* < 0.01. (**E**,**F**) Immunohistochemistry (IHC) showing LC3 (upper panel), CHOP (middle panel), and SESN2 (lower panel) in the tumors from xenograft at a 400× magnification. (Scale bar, 20 μm). **, *p* < 0.01 and ***, *p* < 0.001.

HCT116 Luc^+^ cells (1 × 10^7^ in 100 μL) were subcutaneously injected into five-week-old female nude mice and then treated with 10 mg/kg absolute ethanol (EtOH), 10 mg/kg DHA, 10 mg/kg Oxaliplatin, or a combination treatment (10 mg/kg DHA with 10 mg/kg Oxaliplatin). When the tumor volume reached approximately 200 mm^3^, the mice were randomized into four groups of six mice per group. The Oxaliplatin and DHA combination significantly decreased the intensity of bioluminescence in HCT116 Luc^+^ cells (Figure 6A) and tumor growth compared to EtOH or treatment with Oxaliplatin or DHA alone (Figure 6B,C). Tumor weight was lower for the combination treatment compared to that in other groups (Figure 6D). We performed an immunohistochemistry (IHC) to measure the expression of LC3, CHOP, and SESN2. Consistent with the in vitro results, LC3, CHOP, and SESN expression in the combination treatment group was increased compared to results in the other groups (Figure 6E,F), suggesting that the combination treatment enhanced autophagy via the ER stress-SESN2 pathway.

## 3. Discussion

Oxaliplatin together with 5-FU significantly improves the survival of CRC patients treated with antibodies, such as bevacizumab and cetuximab [27]. However, this treatment causes many side effects. Therefore, combinatorial therapies involving other agents were sought-after to reduce these side effects [28]. The major adverse effects of Oxaliplatin are neurotoxicity, which is induced by the release of proinflammatory cytokines, such as interleukin 6 (IL-6), tumor necrosis factor alpha (TNF-α), and cyclooxygenase 2 (COX-2) [29]. DHA can reduce the expression of proinflammatory cytokines and COX-2 by synthesizing neuroprotection D1 [30]. Therefore, the combination of Oxaliplatin and DHA may reduce Oxaliplatin-induced neurotoxicity**.** Moreover, although several studies have indicated that DHA increased drug sensitivity by generating reactive oxygen species, lipid peroxidation, and the incorporation of the drug into membrane phospholipid and lipid rafts [31,32], the mechanism underlying increased drug sensitivity via autophagy remains unclear. The current study investigated whether DHA synergized with Oxaliplatin in CRC treatment.

Our results showed that the Oxaliplatin and DHA combination did not affect normal colon cancer cells, but it induced a significant decrease in viability in the CRC cell lines. Interestingly, ω6-PUFAs (AA) or other ω3-PUFAs (α-LA and EPA), alone or combination with Oxaliplatin, had no effect on cell viability. Whereas ω6-PUFAs induce inflammation and cancer, ω3-PUFAs exert anticancer effects [33]. Previous studies have indicated that DHA exhibits more potent effects than α-LA and EPA due to its higher degree of unsaturation [14]. Therefore, differences in these effects may be related to the degree of unsaturation.

Interestingly, autophagy induced by rapamycin (autophagy inducer) and CQ (lysosomal inhibitor) significantly increased autophagy when treated with Oxaliplatin and DHA for 24 h, whereas autophagy was not increased by rapamycin for 48 h. Perhaps autophagy would occur too much, resulting in the death of all cells and the lack of autophagic cells, as well as the expression of p62 and LC3.

*SESN2* is a stress-sensitive gene that is expressed in all tissues and regulates cell growth, viability, and metabolism [9]. SESN2 expression is reportedly reduced in cancer tissues compared to normal tissues, and it is lost or downregulated during the carcinogenesis of CRC [25]. Consistent with this, our data showed that both the mRNA and protein expression levels of SESN2 were significantly decreased in the tissues of CRC patients and CRC cell lines, compared to the levels in normal tissues and colon cells. During autophagy, SESN2 increases the recruitment of liver kinase B1 (LKB1) and the subsequent phosphorylation of Thr172 in the catalytic subunit of AMPK. This increases AMPK activity and inhibits mTOR [34]. DHA increased AMPK and decreased mTOR via the Oxaliplatin (data not shown), suggesting that the Oxaliplatin and DHA combination further increased autophagy by elevating LKB1 recruitment.

ER stress is an important factor which regulates SESN2-induced autophagy [35]. ER is an essential organelle that maintains homeostasis by regulating the biosynthesis of membrane proteins and lipids. When this homeostasis is disturbed, chaperone expression in the ER is increased. This is called ER stress [35,36]. ER stress is mediated by 3 ER membrane proteins, IRE1α, PERK, and ATF6, which ultimately regulate the transcription factor CHOP via an intracellular signaling cascade [37]. Reportedly, DHA induces ER stress via ATF6 and PERK activation [38]. However, in our study, DHA did not affect ATF6, but it elevated the expression of CHOP via the PERK cascade. Our data differed from the data of a previous study, in which SESN2 activation was attributed to ATF4 [36,39]. Interestingly, in our system, it was CHOP, and not ATF4 that binds to the promotor region of SESN2 to increase the SESN2 expression in the presence of the Oxaliplatin and DHA combination. Further investigation of this difference in the modulation of SESN2 expression by the Oxaliplatin and DHA combination is warranted, although the phenomenon may be associated with the unique genetic backgrounds of different cancer cell types and the variance in response to drugs.

## 4. Materials and Methods

### 4.1. Cell Lines and Cultures

Human CRC HCT116, DLD-1, HT29, and SW620 cells, as well as human colon CCD-18Co and FHC cells, were purchased from the American Type Culture Collection (ATCC, Manassas, VA, USA) and the HCT116 Luc^+^ Cells were obtained from the Japanese Collection of Research Bioresources Cell Bank (Osaka, Japan). Moreover, all cell lines were not mycoplasma contamination.

The HCT116 and HCT116 Luc^+^ cells were grown in McCoy’s 5A medium (Welgene, Farmingdale, NY, USA), the CCD-18Co cells were cultured in Eagle Minimum Essential Medium (EMEM, ATCC), and the other cell lines were grown in RPMI 1640 medium. All media were supplemented with 10% fetal bovine serum (HyClone, Logan, UT, USA) in a 37 °C humidified chamber with 5% CO_2_.

### 4.2. Reagents and Antibodies

DHA, α-LA, EPA, and AA were purchased from Cayman chemical (Ann Arbor, MI, USA) and then dissolved in EtOH. CQ, rapamycin, and thapsigargin were purchased from Sigma (St. Louis, MO, USA) and stored at −20 °C until further use.

The following antibodies were used in this study; GRP78, GRP94, Bip, CHOP, LC3, and p62. All of the above were purchased from Cell Signaling Technology (Beverly, MA, USA). SESN2 and CHOP (growth arrest and DNA damage 153 (GAD153)) were obtained from Santa Cruz Biotechnology (Paso robles, CA, USA). The secondary antibodies, anti-mouse-IgG-horseradish peroxidase (HRP), were purchased from Santa Cruz Biotechnology, and the anti-rabbit-IgG-HRP was purchased from Cell Signaling Technology.

### 4.3. Cell Viability Assay and Apoptosis

Cell viability was measured using the Cell Viability Assay Kit (EZ-Cytox, DOGEN, Daejeon, Korea). Human CRC HCT116, DLD-1, HT29, and SW620 cells, and normal colon FHC and CCD-18Co cells (1 × 10^4^ cells per well) were seeded on 96-well plates and then treated as described in the Results section. The cells were then incubated with the EZ-Cytox reagent and incubated for 2 h at 37 °C in an atmosphere of 5% CO_2_. Absorbance at 450 nm was determined using a microplate reader (SPECTRA190, Molecular Devices, Sunnydale, CA, USA).

To detect the translocation of phosphatidylserine, a marker of apoptosis, from the inner to the outer leaflet of the plasma membrane, the cells were stained with Annexin V according to the manufacturer’s protocol for the fluorescein isothiocyanate Annexin V Apoptosis Detection Kit (BD Biosciences, San Diego, CA, USA). Flow cytometry was performed using an Accuri C6 flow cytometer (BD Biosciences).

### 4.4. CI Analysis

Combination indexes (CIs) were calculated using the Compu Syn software (ComboSyn, Inc., Paramus, NJ, USA). The extent of synergism/antagonism was determined based on the CI values, where, in general, CI values below 1 suggested synergism, whilst CI values above 1 indicated antagonism between the drugs.

### 4.5. GFP-LC3 Punctuation

Cells were grown to 70% confluence in growth medium for 18 h, prior to infection with recombinant adenoviruses expressing GFP-tagged LC3 (GFP-LC3, a gift from Professor Kyu Lim, Chungnam National University, Korea). Infected cell cultures were then treated with the combinatorial treatment of Oxaliplatin and DHA for 4 h. The cells were observed via confocal microscopy (Carl Zeiss, Oberkochen, Germany).

### 4.6. Immunocytochemistry

The HCT116 cells were seeded on round glass coverslips. Treated cells were fixed with 3.7% formaldehyde for 15 min at room temperature (RT), and then, 0.5% Triton X-100 was used for permeabilization under the same conditions. The cells were blocked with 3% bovine serum albumin for 1 h, and then incubated overnight with primary antibodies, both at 4 °C. Subsequently, 4′,6-diamidino-2-phenylindole (DAPI, Invitrogen, CA, USA) was used to stain the nuclei, followed by incubation with specific secondary anti-bodies for 17 min at 4 °C.

### 4.7. Genomic Data Analysis

The mRNA expression data of the TCGA colon adenocarcinoma cohort was downloaded on 1 March 2019, from the TCGA hub of the UCSC Xena website (https://xenabrowser.net/). From the TCGA hub, the expression and correlation of mRNA were analyzed using 329 patients with colorectal cancer from the IlluminaHiSeq of TCGA Colon Cancer (Colon Adenocarcinoma). The mRNA dataset was calculated as log2 (x + 1) and normalized to RNA-Seq by Expectation-Maximization. Amongst the 329 data, 327 samples of the patients with ‘primary tumor’ and ‘solid tissue normal’ were analyzed and then 286 primary tumors were used for correlation analysis. R version 3.5.2 was used as the statistical analysis software.

### 4.8. Immunoblotting Assay

Western blotting was carried out as previously described in Reference [40].

### 4.9. Transfection

HCT116 cells were transfected with SESN2 siRNA and CHOP small interfering RNA (siRNA) that were purchased from Santa Cruz Biotechnology (Santa Cruz). To warm the cells prior to siRNA treatment, the dish was treated with Opti-MEM (Gibco, Life Technologies, Rockville, MD, USA) and then incubated at 37 °C for 30 min. Next, the mixture containing siRNA, Opti-MEM, and Lipofectamine RNAiMAX (Invitrogen, Carlsbad, CA, USA) was incubated for 30 min at RT. The mixture was then added to the warmed cells and then incubated for 6 h at 37 °C in a 5% CO_2_ incubator and changed media. Then, the cells were treated with DHA and Oxaliplatin for analysis.

### 4.10. Isolation of RNA and Quantitative Real-Time Polymerase Chain Reaction (qRT-PCR)

Total RNA was extracted using the TRIzol reagent (Life Technologies, Rockville, MD, USA), according to the manufacturer’s instructions. Then, qRT-PCR was performed using Applied Biosystems™ QuantStudio™ 6 Flex Real-Time PCR (Thermo Fisher Waltham, MA, USA).

### 4.11. ChIP Assay

The ChIP assay was performed as previously described in reference [41]. Isolated DNA was amplified via PCR using the following specific primers: P1 forward, 5′ TAC TCA GCT CTG TTC TCA CAC ACA 3′; reverse, 5′ GTT AGC CAG GAT GGT CTT CAT CT 3′ and P2 forward, 5′ GTA GGC ACC TTT CTT GAA CAC C 3′; reverse, 5′ ACC TCC ACC GTT CTA TTG TGA G 3′, P3 forward, 5′ CAG TAA AGG CTC TCC TAC AAA GAG G 3′; reverse, 5′ CCT ATG CAG AAA CTC CAC T 3′ and P4 forward, 5′ CAA CAG AGC AAG ACT CCG TCT AA 3′; reverse, 5′ GCA GAT TCC ATG AGA AAC TAC CTG 3′.

### 4.12. Autophagic Flux

The autophagic flux was measured using an autophagy assay kit (Abcam, Cambridge, UK). The cells were seeded in a 6-well plate, and the next day, rapamycin and CQ were treated for 1 h before the Oxaliplatin and DHA treatment and then incubated for 24 h and 48 h. The harvested cells were stained with green detection reagent at 37 °C and then analyzed by flow cytometry.

### 4.13. Xenograft

All the experiments involving live mice were performed according to the Guidelines and Regulations of the Korea University Institutional Animal Care and Use Committee (IACUC, KOREA-2018-0084). The mice used were 4–week-old female BALB/c nude mice purchased from the Orient Bio (Seongnam, Korea). The animals were acclimated for 1 week prior to the study and they were provided with free access to food and water. HCT116 Luc^+^ cells (1 × 10^7^), in 100 μL of phosphate-buffered saline, were subcutaneously implanted into the mice. The tumor size was measured every 2 days and then simultaneously calculated. When the tumor volume reached approximately 200 mm^3^, the mice were randomized into four groups of six mice per group. Every 2 days, DHA and Oxaliplatin were intraperitoneally and hypodermically injected, respectively. The mice were euthanized when the tumor size and overall health status were met by euthanasia criteria. The volume was calculated as 0.5 × length × (width)^2^. 

### 4.14. IHC

Paraffin sections were deparaffinized in xylene and rehydrated via a descending ethanol concentration gradient. Antigen epitopes were unmasked using a sodium citrate buffer (pH 6.0). Subsequently, the sections were incubated overnight at 4 °C with the following primary antibodies: SESN2, LC3, and CHOP. Then, sections were incubated with the appropriate secondary antibodies, followed by treatment with freshly prepared 3′-Ddabiaminobenzidine substrates. Sections were lightly counter-stained with hematoxylin and then mounted.

### 4.15. Statistical Analysis

All the experiments were conducted respectively and repeated a minimum of three times. Statistical analysis was carried out using the GraphPad Prism 6 software (GraphPad Software, Inc., San Diego, CA, USA). The results were expressed as the mean of arbitrary values ± SEM. All results were evaluated using the unpaired Student’s t test, in which a *p*-value of less than 0.05 was considered significant (*, **, and *** means *p* < 0.05, *p* < 0.01, and *p* < 0.001, respectively).

## 5. Conclusions

**Figure 7:**
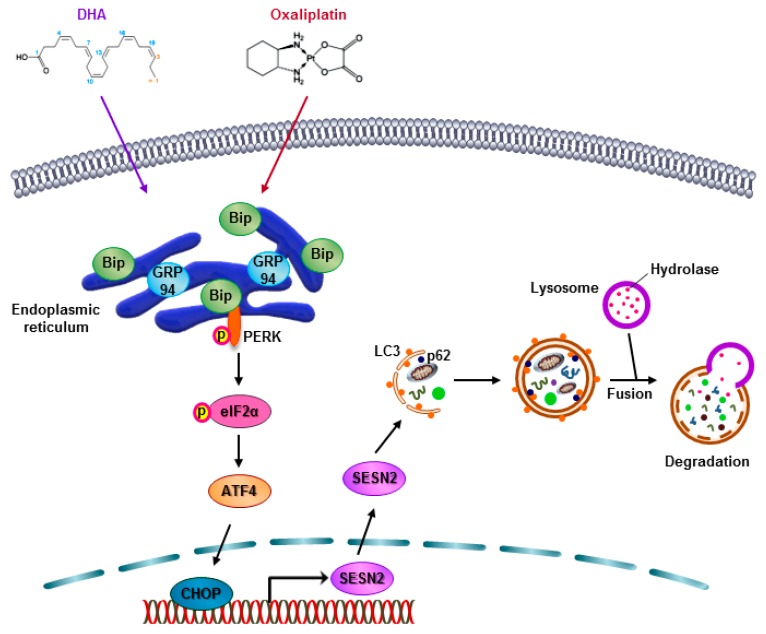
Scheme of autophagic cell death induced by the combination of Oxaliplatin and DHA.

In summary, the Oxaliplatin and DHA combination induced autophagic cell death in the CRC cells. This promotion of autophagy was due to ER stress overproduction and the subsequent increase in SESN2 activity (Figure 7). Combination therapies using natural products and nutritional supplements, aimed at reducing the side effects of existing anticancer drugs, are currently undergoing clinical trials. The current study focused on the effect of an Oxaliplatin and DHA combination on CRC and the findings indicated that Oxaliplatin reduced neurotoxicity, thereby showing potential as a new therapeutic strategy for CRC. 

## Supplementary Materials

The following are available online at https://www.mdpi.com/2072-6694/11/7/982/s1, Figure S1: Other ω3-PUFAs or ω6-PUFAs have no effect with Oxaliplatin, Figure S2: DHA enhances Oxaliplatin-induced autophagy, Figure S3: Combinatorial treatment with DHA and Oxaliplatin reduces viability and results in SESN2-mediated autophagy in PDC cells.
