# Supplementary material for: Docosahexaenoic Acid Enhances Oxaliplatin-Induced Autophagic Cell Death via the ER Stress/Sesn2 Pathway in Colorectal Cancer
Source: Cancers (Basel). 2019 Jul 14;11(7):982. doi: 10.3390/cancers11070982 (PMC6678695; doi:10.3390/cancers11070982)
Supplement: Supplementary file 1 [file cancers-11-00982-s001.pdf]

Supplementary Materials: Docosahexaenoic acid enhances Oxaliplatin-induced autophagic cell death via the ER stress/SESN2 pathway in colorectal cancer

Soyeon Jeong, Dae Yeong Kim, Sang Hee Kang, Hye Kyeong Yun, Jung Lim Kim, Bo Ram Kim, Seong Hye Park, Yoo Jin Na, Min Jee Jo, Yoon A Jeong, Bu Gyeom Kim, Dae-Hee Lee and Sang Cheul Oh

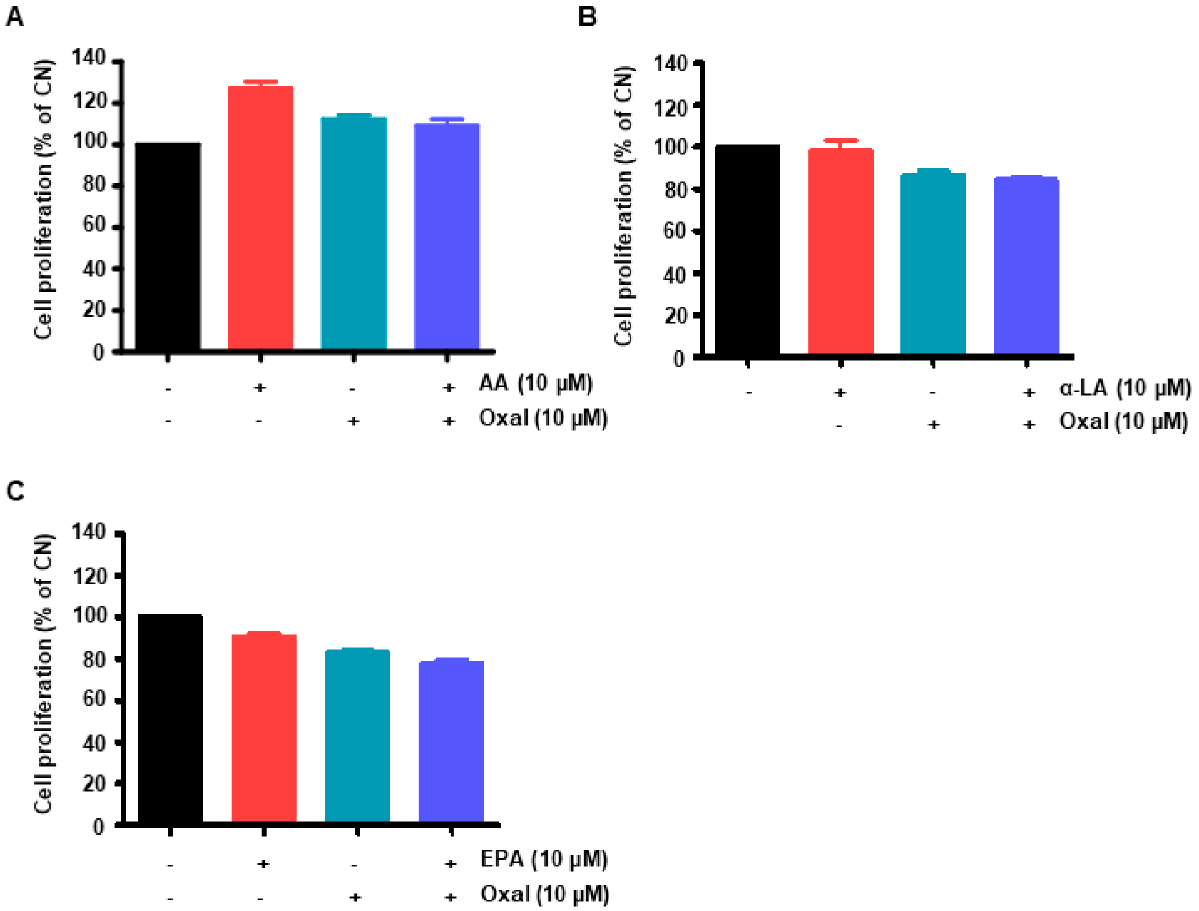

Figure S1. Other ω3-PUFAs or ω6-PUFAs have no effect with Oxaliplatin. (**A-C**) Cell viability was measured by WST-1 after treatment with 10 μM Oxaliplatin and 10 μM ω6-PUFAs arachidonic acid (**A**) or ω3-PUFAs alpha-linolenic acid (**B**) and eicosapentaenoic acid (**C**).

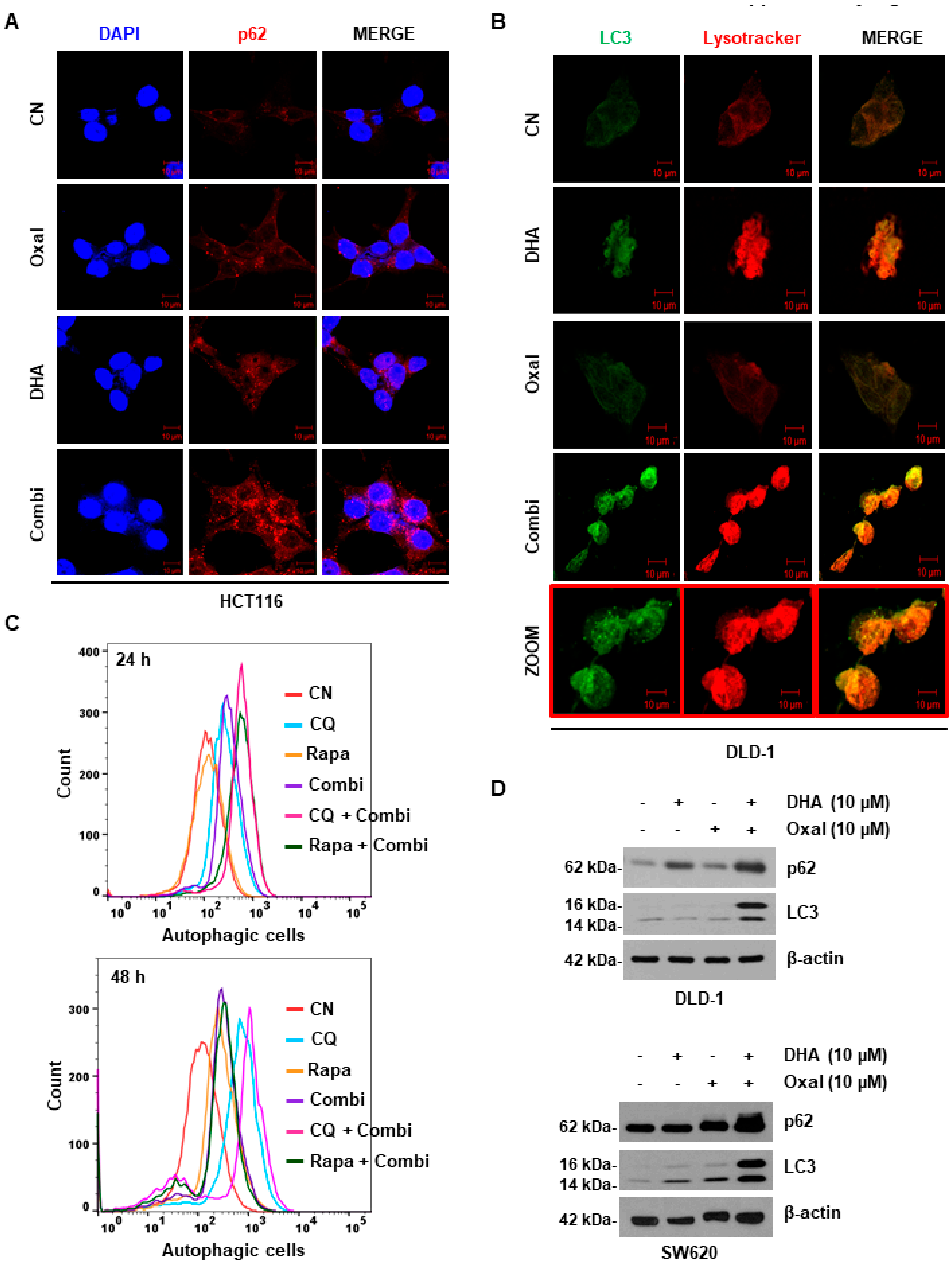

Figure S2. DHA enhances Oxaliplatin-induced autophagy. (**A**) HCT116 cells were treated with Oxaliplatin and DHA treatment and p62 expression was observed. (**B**) Formations of GFP-LC3 puncta following Oxaliplatin and DHA treatment were analyzed via confocal microscopy (Scale Bar, 10 μm). (**C**) HCT116 cells were exposed to Oxaliplatin and DHA with or without CQ or rapamycin for 24, and 48 h. The autophagic cells were analyzed by flow cytometry and quantified. (**D**) The protein levels of p62 and LC3 were evaluated by western blotting in DLD-1 (upper) and SW620 (lower) cells.

A

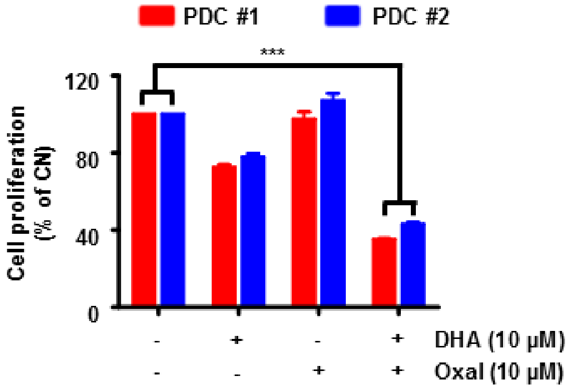

B

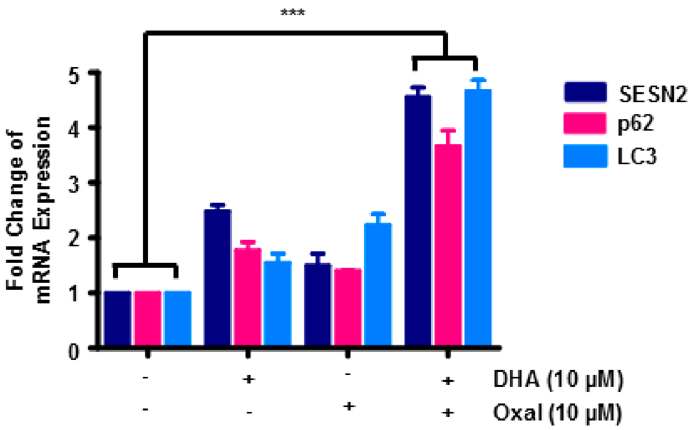

Figure S3. Combinatorial treatment with DHA and Oxaliplatin reduces viability and results in SESN2-mediated autophagy in PDC cells. (**A**-**B**) PDC cells were exposed to Oxaliplatin and DHA, and analyzed via WST-1 assay (**A**), and qRT-PCR (**B**). ***, $p < 0.001$.

Figure 2A.

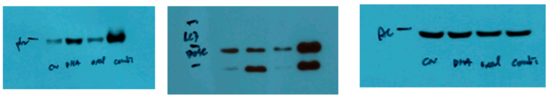

Figure 2B.

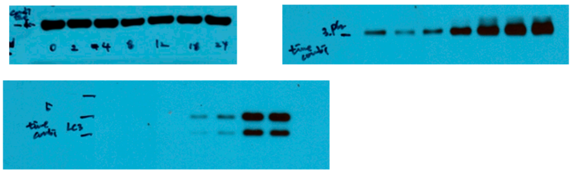

Figure 2F.

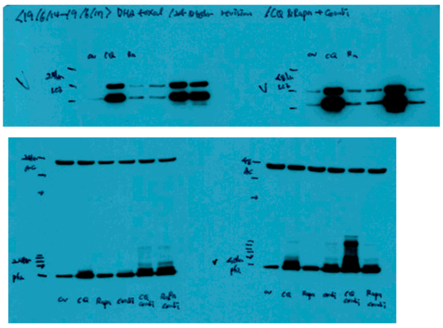

Figure 3B. SESN2, actin

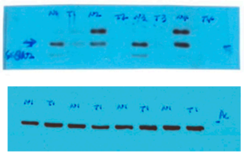

Figure 3E. SESN2, actin

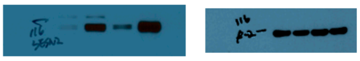

Figure 3H. SESN2, p62, LC3, actin

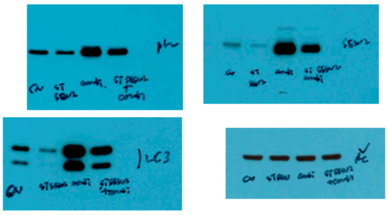

Figure 4A. 원본

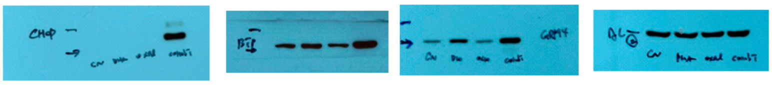

Figure 4B. 원본

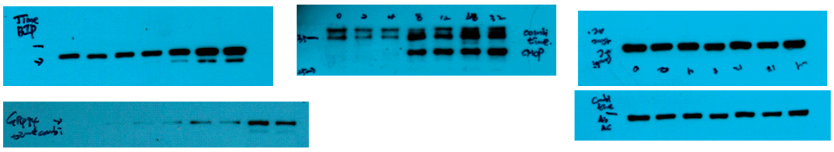

Figure 4E. 원본

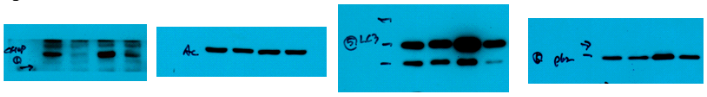

Figure 4F. 원본

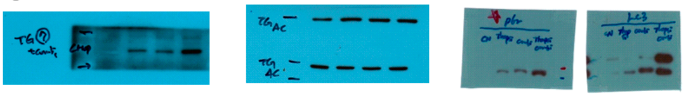

Figure 5B

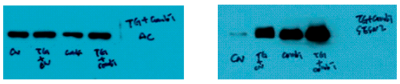

Figure 5D

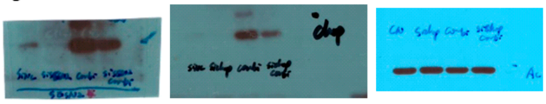

Figure 5F

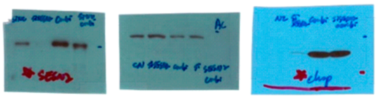

Supplementary Figure 2D.

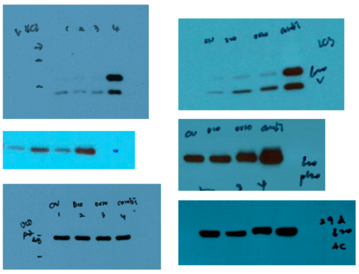

The densitometry readings/intensity ration of all western blot bands. Unfortunately, we do not have a whole blot showing all molecular weight markers because we cut a single membrane to identify expression of several molecules. So we only have a fragmented band film with a molecular weight.

© 2019 by the authors. Licensee MDPI, Basel, Switzerland. This article is an open access article distributed under the terms and conditions of the Creative Commons Attribution (CC BY) license (http://creativecommons.org/licenses/by/4.0/).
